# Assessment of peri-implant bone stress distribution with the effect of attachment type and implant location using finite element analysis

**DOI:** 10.34172/joddd.2023.40483

**Published:** 2023-12-30

**Authors:** Shima Aalaei, Atefeh Sheikhi, Parisa Mehdian, Farnoosh Taghavi, Sara Salimian, Farnaz Taghavi-Damghani

**Affiliations:** ^1^Department of Prosthodontics, Dental Caries Prevention Research Center, Qazvin University of Medical Sciences, Qazvin, Iran; ^2^Student Research Committee, Qazvin University of Medical Sciences, Qazvin, Iran; ^3^Department of Prosthodontics, School of Dentistry, Shahid Beheshti University of Medical Sciences, Tehran, Iran; ^4^Student Research Committee, Tehran University of Medical Sciences, Tehran, Iran; ^5^Dental Caries Prevention Research Center, Qazvin University of Medical Sciences, Qazvin, Iran

**Keywords:** Dental implants, Finite element analysis, Overdenture, Stress

## Abstract

**Background.:**

The objective of the current research was to evaluate how stress is distributed in the peri-implant bone of a mandibular overdenture with implants placed asymmetrically to the midline.

**Methods.:**

A 26-year-old male’s mandible, with missing teeth, was examined using computed tomography (CT) scanning. Two implants were inserted at right angles to the occlusal plane, in the positions of the right canine and left lateral incisor of the mandible, with an internal connection. Two types of attachments (bar and ball) were designed. To simulate the clinical condition, anterior (on central incisors) and bilateral posterior (on premolars and molars) loadings were applied. The stress distribution was assessed using finite element analysis (FEA).

**Results.:**

The lateral incisor level implant was found to have the highest maximum principal stress (about 33 MPa) in both models in the anterior loading condition. However, in both models, the canine-level implant revealed more stress values (about 22 MPa) in the posterior loading condition.

**Conclusion.:**

In mandibular implant-supported overdentures, when implants were placed asymmetrically to the midline, one acted as a fulcrum and sustained more occlusal load. The bar attachment system did not reveal superior results in terms of stress distribution compared to the ball attachment.

## Introduction

 Implant-supported overdentures have been introduced to provide esthetics and functional rehabilitation for patients wearing complete dentures.^1,2^ The high success rate of dental implants has led to the selection of an overdenture based on two implants as one of the treatment options in the edentulous mandible.^3^

 There are splinted (bar attachments) and non-splinted (ball, locator, etc.) anchorage systems that can improve the retention and stability of an implant-supported overdenture.^4^ Attachment selection is one of the challenges among clinicians and depends on multiple factors such as retention, occlusal space, and jaw anatomy.^5^

 Attachments transfer the stress from mastication to the implants, and clinicians should consider the stress values to be in a safe range.^6^ Ball attachments are simple to use and cost-effective and can reduce the occlusal force by absorbing the loading stress. Patients can clean this type of attachment easily.^5,7^ More vertical restorative space is needed for the bar and clip attachment compared to the non-splinted ones.^5^ The stress values can be reduced using the bar attachment, as suggested by Misch,^8^ due to the splinting ability.

 Finite element analysis (FEA) is one of the methods to assess stress distribution in bone‒implant systems. It has several advantages, such as generating complex models and analyzing internal stress accurately.^9,10^

 The failure of an implant system is greatly influenced by the level of stress concentration in the peri-implant bone.^6^ Misch^8^ suggested that the loading forces would be reduced by placing the implants at the same occlusal height and symmetrically from the midline. A few studies have evaluated the stress distribution in models with different implant positions.^11,12^ Alvarez-Arenal et al^3^ reported that implants at the premolar level had better stress distribution than implants at the lateral incisor and canine levels. No evidence was found to compare asymmetrical implants from midline with different types of attachments. Hence, the innovation of this study is that the effect of two parameters with different loading conditions was analyzed. This study assessed and compared stress distribution in the bone adjacent to the implants placed asymmetrically from the midline with ball and bar attachments.

## Methods

 A study model was created using a computed tomography (CT) scan of a 26-year-old man’s edentulous mandible to evaluate how stress is distributed in the bone around the dental implants.

**Figure 1:**
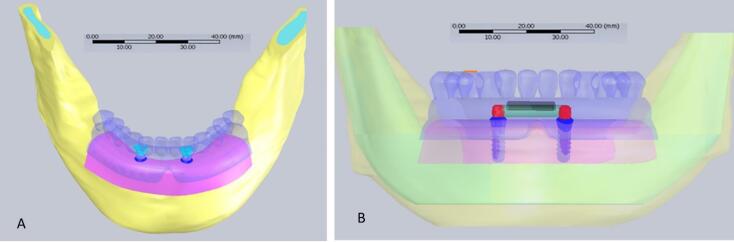
3D models of the A) bar and B) ball attachments

**Figure 2:**
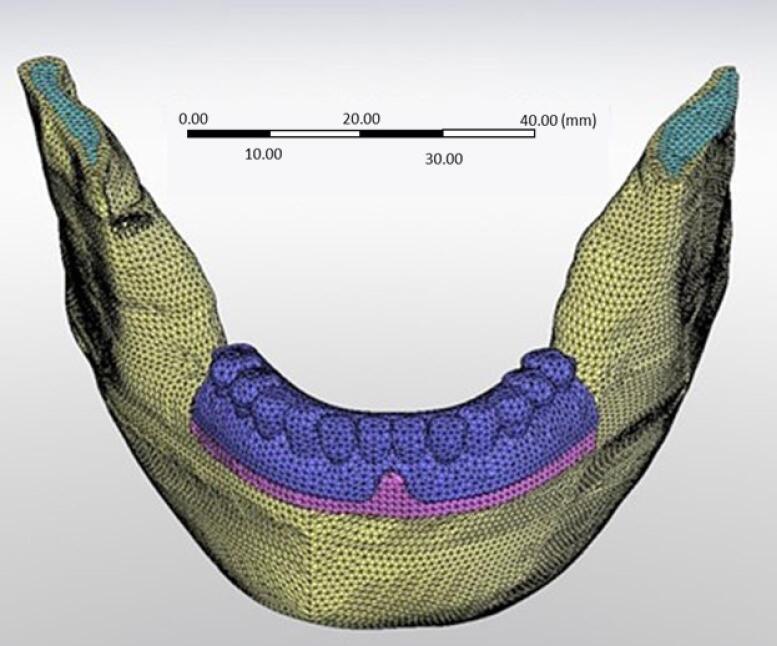
Meshing process

 Data were processed with an image processing software, MIMICS (Materialise Interactive Medical Image Control System; Materialise, version 21, Leuven, Belgium). Then, the image was transferred to SolidWorks software (version 28, Dassault Systems SolidWorks Corp., MA, United States). Two bone-level implants (ITI, Straumann, Switzerland, 10 × 4.1 mm) were modeled and placed in the right canine and left lateral incisor region. The Dolder bar (3.25 mm height) and ball attachments (3.4 mm height) were inserted in two separate models (Figure 1). The bone thickness was extracted from the CT scans, and a mucosal membrane with a 2-mm thickness was modeled using SolidWorks software. All the simulated materials were considered homogeneous with a linear modulus of elasticity. The final model was meshed, taking into account the physical properties of the materials (Table 1) and the boundary conditions (Figure 2). The ball model had 468 472 elements, while the bar model had 150 566 elements.

**Table 1:** Physical properties of the materials

**Materials**	**Elastic modulus (MPa)**	**Poisson ratio**	**Reference**
Cortical bone	13700	0.3	^3,13^
Acrylic resin	3000	0.35	^14^
Trabecular bone	1370	0.3	^3^
Mucosa	680	0.45	^14^
Ball abutment and metallic cap	114000	0.3	^3^
Implant	110000	0.33	^15^
Bar	218000	0.33	^16,17^
Clips	3000	0.28	^18^
Lamella retention insert	97000	0.42	^6^

 The total number of nodes in the bar model was 223 173, and in the ball model was 746 557. The size of each element was 1.5 mm. Parabolic and tetrahedral solid elements were used.

**Figure 3:**
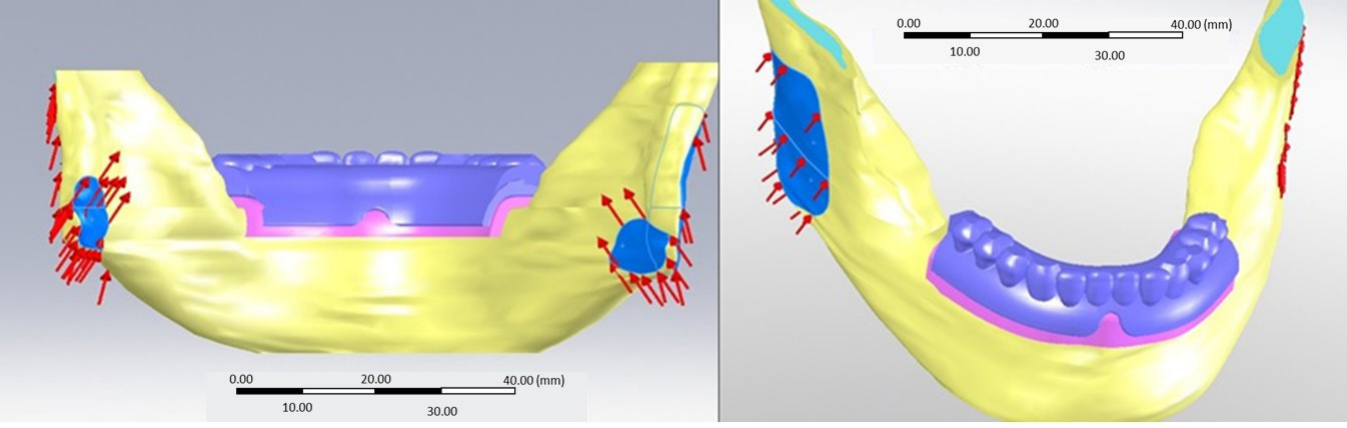
Modeling of the muscles

**Figure 4:**
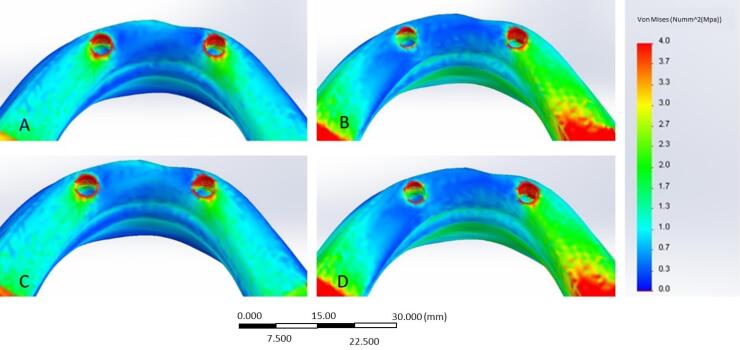
Stress values in the A) bar model - anterior loading, B) bar model - bilateral posterior loading, C) ball model - anterior loading, and D) ball model – bilateral posterior loading

 The COSMOWorks software (version 12.1., Dassault Systems SolidWorks Corp., MA, United States) was used to model the masseter and internal pterygoid muscle and apply a loading condition on the anterior (central and lateral incisors) and posterior (molars and premolars) regions (Figure 3). The amount of muscle force in each condition was based on the studies of Korioth and Hannam^13^ and determined by the multiplication of two parameters, namely weight factor and scaling factor (Table 2). The stress values were analyzed and described in color-coded figures (Figure 4).

**Table 2:** The forces of the muscles (weight factor and scaling factor)

	**Weight factor (Newton)**	**Scaling factor**			
		**Anterior clenching**		**Posterior clenching**	
		**Right**	**Left**	**Right**	**Left**
Superficial masseter	190.4	0.40	0.40	1.00	1.00
Deep masseter	81.6	0.26	0.26	1.00	1.00
Medial pterygoid	174.8	0.78	0.78	0.76	0.76

## Results

 In the present study, the stress distribution in the peri-implant bone was measured using FEA in different loading conditions (Table 3). In the bar model, by applying anterior loads, the cortical bone of the lateral incisor-level implant exhibited the maximum stress (33.3 MPa). The canine-level implant showed a stress value of 22.7 MPa in this loading condition. The canine-level implant had the highest maximum principal stress (22 MPa) by bilateral posterior loading application. The cortical bone surrounding the lateral incisor-level implant had a stress value of 20.7 MPa.

**Table 3:** Maximum stress values (MPa) with bar and ball attachments

		**Anterior loading**	**Posterior bilateral loading**
Bar attachment	Right implant (canine)	22.7	22
	Left implant (lateral incisor)	33.3	20.7
Ball attachment	Right implant	27.4	22.2
	Left implant	33.2	17.9

 The cortical bone of the implant at the level of the lateral incisor had the highest principal stress (33.2 MPa) when anterior loads were applied in the ball model. The adjacent bone stress value at the canine-level implant measured 27.4 MPa.

 The stress value in the bone adjacent to the canine-level implant was 27.4 MPA. Under bilateral posterior clenching, the maximum principal stress was 22.2 MPa in the canine-level peri-implant bone, and 17.9 MPa was recorded adjacent to the lateral incisor-level implant. Therefore, the distal implant exhibited the highest stress concentration in both models when subjected to posterior loads, and with anterior loads, the medial implant in both models exhibited more stress.

## Discussion

 In this study, the stress was analyzed in the bone adjacent to the asymmetrically placed implants. No similar study was found in the literature to compare the results. Studies in biomechanics have demonstrated that the primary cause of crestal bone loss and implant failure shortly after the implant is loaded is primarily due to a great deal of stress at the implant‒bone interface.^18,19^ In the present study, the greatest stress concentration was found in the cortical bone surrounding the implant neck due to the higher density and modulus of elasticity of cortical bone compared to that of trabecular bone.

 The findings regarding the use of splinted or non-splinted attachments have controversies in different articles.^8,20,21^ Misch^8^ suggested that the bar attachment can distribute the stress more evenly compared to the ball attachment. Satpathy et al^20^ showed that ball attachment could be a favorable system in conditions with a low range of force. However, a bar/clip attachment may have better results when a higher force range is expected. Park et al^21^ assessed the effect of attachment type and palatal coverage in the maxillary implant-supported overdentures. They concluded that ball attachment revealed better stress distribution than that of the attachment of the milled bar.

 In this study, both models showed similar stress levels in the peri-implant bone adjacent to the area where the load is applied. However, in the peri-implant bone far from the place of load application with anterior loads, the model with the bar attachment showed less stress. With posterior loads, the model with ball attachments exhibited lower stress values due to the rotational movements around the ball attachments.

 According to the present study, the bar attachment system did not reveal superior results in stress distribution compared to the ball attachment in asymmetrically placed implants. The ball attachment allows a wide motion range for the prosthesis and absorbs stress. This free-rotating motion of overdenture increases the force distribution in the mucosal tissue and reduces the stress accumulation in the implant and the surrounding bone.^22^ Bar attachment splint fixtures increase the retention and reduce the range of motion.^8,23^ Therefore, the prosthesis has limited anteroposterior movements with bilateral posterior loads. In this study, it can be discussed that with anterior loads, the labial part of the anterior alveolar bone in both models prevented the continued rotation of the prosthesis. Hence, the effect of bar attachment in terms of stress distribution was greater than the ball attachment.

 Misch^8^ described that implants should be placed symmetrically from the midline. When one implant is farther from the midline, it will act as the rotation point during posterior load application. However, the anterior implant will act as a fulcrum and show higher stress in the anterior bite condition, consistent with the present study.

 FEA is a numerical method with great efficiency in stress distribution analysis. This method has the advantage of simulating models with complex geometries and the possibility of changing mechanical parameters. Modeling of trabecular and cortical bone to assess the stress patterns is difficult due to the heterogeneous structure and several factors such as age, gender, and type of the bone.^12,23,24^ In this study, the mechanical properties of the bone were assumed to be isotropic and homogeneous, based on the methods used in several previous research.^12,24^

 The data obtained from this study provide information on the precise areas where stress is concentrated. However, it is necessary to conduct long-term clinical studies to determine the exact stress values and the effect of loadings on the surrounding tissues.

## Conclusion

 According to the results, better stress distribution was not achieved by using splinted attachments compared to the non-splinted attachments. The model with the bar attachment showed less stress in the peri-implant bone far from the place of load application with anterior loads, and the model with ball attachments exhibited lower stress values due to the rotational movements around the ball attachments with posterior loads. When implants were placed asymmetrically in the mandible, one implant acted as a fulcrum after applying occlusal loads. The distal implant exhibited more stress values under posterior loading; the medial implant showed greater stress values under anterior loads.

## Competing Interests

 The authors declare that they have no conflicts of interest.

## Data Availability Statement

 The data used to support the findings of this study are included in the article.

## Ethical Approval

 This study was approved by the Ethics Committee of Qazvin University of Medical Sciences with an ethical number of IR.QUMS.REC.1394.682. There was no conflict with ethical considerations.

## Funding

 This research received no specific grant from funding agencies in the public, commercial, or not-for-profit sectors.
